# Evaluation of MEDAG gene expression in papillary thyroid microcarcinoma: associations with histological features, regional lymph node metastasis and prognosis

**DOI:** 10.1038/s41598-019-41701-4

**Published:** 2019-04-09

**Authors:** Yang Song, Li-jun Fu, Hong-ting Li, Xin-guang Qiu

**Affiliations:** 1 Department of Thyroid Surgery, The First Affiliated Hospital of Zhengzhou University, Construction of Road No. 1, Zhengzhou, P. R. China; 2 Department of Oncological Surgery, Xinyang Cental Hospital, Siyi of Road No. 1, Xinyang, P. R. China

## Abstract

Papillary thyroid microcarcinoma accounts﻿ for a large proportion of papillary thyroid carcinoma, especially among new cases. Many  PTMC patients have regional lymph node metastasis, with some experiencing recurrence and even death. However, the risk factors and mechanism by which PTMC relates to these factors are unknown. In this study, differentially expressed genes were identified with microarray from The Cancer Genome Atlas, followed by analysis using the Kyoto Encyclopedia of Genes and Genomes. Immunohistochemistry, immunofluorescence, western blot and Oil Red O staining were carried out to evaluate expression levels and functional alterations. Mesenteric Estrogen Dependent Adipogenesis expression was observed in almost all cases of papillary thyroid microcarcinomas, and the location of expression was associated with histological subtype. High expression was correlated with metastasis and poor disease-free survival. Furthermore, the enrichment analysis indicated that Mesenteric Estrogen Dependent Adipogenesis expression may be associated with metabolic reprogramming to influence metastasis and prognosis. These findings contribute to a better understanding of how Mesenteric Estrogen Dependent Adipogenesis affects metastasis and the prognosis of papillary thyroid microcarcinoma patients and suggest that Mesenteric Estrogen Dependent Adipogenesis expression may be a novel prognostic marker in these patients.

## Introduction

Papillary thyroid microcarcinoma (PTMC) is defined as a papillary thyroid carcinoma (PTC) not more than 1 cm, accountings for the majority of new cases of PTC. PTC is the most common malignancy of the endocrine system, with a rapid increase incidence in recent years^1–6^. Clinically, most of PTMCs are distinct indolent and follow a rather benign course with good prognosis, while a small portion of cases are characterized by aggressive disease. The clinical management of PTMC is thus a source of significant debate.

In clinical practice, PTMC is usually detected by ultrasound as a thyroid nodule with distinct ultrasonic characteristics. However, ultrasound examination is not ideal for the detection of the lymph node metastasis, which may influence the surgical margins and TNM staging directly. Previous studies showed that approximately 29.0–58.2% of PTMC patients had regional lymph node metastasis (LNM), some of which could not be detected before surgery, with some patients experiencing lymph node recurrence^7–9^. PTMC can be diagnosed by means of fine needle aspiration(FNA), combined with detection of genetic alterations  of genes, such as BRAF, KRASand NRAS, but no single molecular aberration has been proven to be useful in directing PTMC treatment to affect clinical outcomes at present^10–16^.

According to the most recent guidelines, active surveillance may be considered as a substitute for immediate surgery in PTMC patients without clinically evident metastases or local invasion^17^. However, these guidelines also emphasize that additional studies are needed to identify the molecular abnormalities that may distinguish the small number of PTMC patients destined to develop clinically significant disease progression^18^. In short, clarification of the biomarkers and hallmarks associated with poor outcome is urgently needed to further guide PTMC treatment.

To identify the gene expression changes related to the aggressive characteristics of PTMC and its progression, we downloaded the microarray data from TCGA (The Cancer Genome Atlas: https://cancergenome.nih.gov/) and the corresponding clinical data. We found that high expression of Mesenteric Estrogen Dependent Adipogenesis (MEDAG) may be associated with LNM and poor prognosis (p < 0.05, fold change >2). MEDAG, also known as Meda4, is located at 13q12.3 and acts as a regulator of the transcription factor peroxisome proliferator activated receptor gamma (PPARG). Previous investigations suggest that MEDAG overexpression promotes adipogenesis and enhances glucose uptake^19^, and this gene is also significantly differentially expressed in healing gingiva^20^.

## Materials and Methods

### Collection of TCGA public data

RNAseq datasets and clinical data were downloaded from the TCGA database. Gene expression data from 492 PTC samples were downloaded from cBioPortal for Cancer Genomics (https://www.cbioportal.org). A total of 76 patients were included in this study and were each divided into experimental group and control group according to aggressive characteristics (extrathyroid invasion (ETE) or LNM)or poor outcome (recurrence or lethal). RNAseq dataset comparisons between the two groups were performed to find differentially expressed genesusing Student’s t-test. Then Venn diagrams were generated to identify the differentially expressed genes in both the aggressive characteristics group and poor outcome group.

### Patients and Thyroid tumors

In total, 67 patient specimens were gathered from December 2014 to December 2015 at the First Hospital Affiliated to ZhengZhou University and diagnosed in the pathology department; BRAFv600 mutation status was detected by qPCR near the time of collection. All surgically resected specimens were fixed in 4% paraformaldehyde. Among the 67 PTMC patients, 57 had undergone total thyroidectomy, and 10 patients had undergone lobectomy with no evidence of LNM during surgery. Total thyroidectomy and simultaneous neck lymph node dissection was performed in 35 patients with suspicious neck LNM on ultrasonic or surgical exploration. Postoperative radioactive iodine (RAI) remnant ablation therapy was conducted after (T4) withdrawal in patients who showed regional lymph node involvement and extrathyroidal invasion. Higher dose RAI therapy was administered when distant metastasis was detected in whole-body scanning post-therapy. There were 29 patients who received RAI therapy. Stages were determined according to the seventh edition of the tumor node metastasis staging system recommended by the American Joint Committee on Cancer. Clinicopathologic parameters such as age at diagnosis, sex, tumor size, extrathyroidal invasion, and presence of lymph node or distant metastasis were obtained from patients’ medical records. The follow-up time ranged from 1 to 32 months with a median follow-up time of 15 months. Then, an additional 14 PTMC and 4 nodular goiter tissue samples were collected from February to April in 2017 and stored under liquid nitrogen for western blot (WB) and  Oil Red O (ORO) staining. Mesenteric specimens were collected from 3 patients (female, age> 60 years) who underwent unrelated colon operations as positive controls for immunohistochemistry (IHC) and ORO. Permission to use tumor or normal tissues and paraffin slides was granted by all the patients, in accordance with regulations of the Hospital Human Ethics Committee.

### Immunohistochemistry and immunofluorescence staining procedure

IHC staining method was used to detect MEDAG expression in different pathological types of thyroid tissues in 3–µm-thick paraffin sections. Briefly, the slides were first dewaxed in dimethyl benzene, then rehydrated through a gradient of 100%, 95%, 75% and 50% ethanol in water, with antigen retrieval performed in 0.01% sodium citrate solution at 98 °C for 20 min. The slides were treated with 3% H_2_O_2_ in methanol to eliminate endogenous peroxidase activity and blocked with normal goat serum for 40 min at room temperature to prevent nonspecific binding; then, the primary antibody was added and incubated at 4 °C overnight. The next morning, the slides were incubated at 37 °C for 40 min, then washed with PBS and incubated with the secondary antibody (PV-6000 polymer detection system, ZsBio Ltd., China) at 37 °C for 2 h. A color substrate solution from the DAB Detection Kit was applied for 3 minutes, followed by rinsing with tap water for 15 min and then counterstaining with hematoxylin for 15 seconds. Finally, the slides were dehydrated in an ethanol gradient and sealed with neutral gum. Negative controls were included by omitting the primary antibody. Visceral fat depot specimens were used as positive controls. The subcellular localization of the staining and the percentage of stained cells were noted. Immunostaining was blinded and semiquantitatively evaluated in terms of staining intensity (0, negative/−; 1, weak/+; 2, intermediate/++; 3, strong/+++) and percentage of stained cells (0, 0–5%; 1, 5–25%; 2, 25–50%; 3, 50–75%; 4, >75%). An IHC score was calculated by multiplying the proportion of positive cells by the intensity of the staining, with 12 being the maximum score. The cellular localization was also evaluated as cytoplasmic and/or nuclear. All the sections were assessed by two experienced pathologists. In addition, 7 cases of PTMC with inconsistent IHC results were reviewed again by the two pathologists to obtain the final pathological diagnosis. All stained sections were observed under an optical microscope by two experienced pathologists. Immunofluorescence (IF) was used only for special category stain.

For  IF procedure, fluorescein-conjugated secondary antibody was substituted for the PV-6001 polymer detection system, with incubation at 37 °C for 2 h, and the slides were counterstained with DAPI (Beyotime, China) for 60 s.

### Western blot analysis

Protein expression of MEDAG was evaluated by WB. Total protein was extracted from the paracancer, nodular goiter, and tumor tissues using radioimmunoprecipitation (RIPA) lysis buffer with 1% PMSF (Solarbio, China). The protein concentrations were then determined by measuring the absorbance at 280 nm (A280) (NanoDrop 2000C spectrophotometer, ThermoFisher scientific, US). Equal amounts of proteins were loaded, separated using 12% SDS-PAGE, and transferred to a nitrocellulose membrane (Bio-Rad, USA), which was then blocked for 2 h at room temperature in 5% bovine serum albumin. Subsequently, blots were washed and incubated overnight at 4 °C in Tris-buffered saline containing 0.05% Tween 20 (TBST) with a 1:1000 dilution of the first antibody. Membranes were washed three times with TBST and incubated with secondary antibody at a 1:2000 dilution for 1.5 h at room temperature and then washed three times with TBST. Protein expression was detected by an enhanced chemiluminescence (ECL) method and imaged with a FluorChem Systems instrument (Proteinsimple, USA).

The following antibodies were used in IHC, IF and WB. Primary antibodies: rabbit anti-MEDAG (Signalway Antibody LLC., USA.), mouse anti-β Tubulin (ZsBio Ltd., China), mouse anti-PPARG (Proteintech Group, China). Secondary antibody: fluorescein(FITC)- conjugated goat anti-rabbit IgG (ZsBio Ltd., Beijing, China), TRITC- conjugated goat anti- mouse IgG (ZsBio Ltd., China), goat anti-rabbit IgG HRP conjugated (Signalway Antibody LLC., USA.), HRP-conjugated affinipure goat anti-rabbit IgG (Proteintech Group, China).

### Oil Red O staining procedure

Oil Red O (ORO) was performed conventionally. Briefly, frozen PTC and para-cancer tissues were cryosectioned at 10 μm thickness on a freezing microtome (CRYOSTAR NX50, Thermo, China) and stained with Oil Red O(Servicebio, China) for 10 min. After rinsing in distilled water, sections were counterstained for 5 min in hematoxylin. The percentage of ORO-stained cells was determined (0, 0–5%; 1, 5–25%; 2, 25–50%; 3, 50–75%; 4, >75%).

### Differentially expressed gene analysis

Enrichment analysis was carried out by KEGG Mapper^21^ (https://www.genome.jp/kegg/mapper.html).

### Statistical analysis

With SPSS 21.0, the statistical significance of categorical data was determined by χ^2^ tests or Fisher’s exact test. The Wilcoxon rank sum test was used to compare two sets of data. Survival was analyzed by the Kaplan–Meier method and compared by the log-rank test. The odds ratio (OR) and the confidence interval (CI) for the relationship between MEDAG expression and disease free survival (DFS) were calculated. The survival analysis was based on disease-free survival. Survival time was calculated as the time from the beginning of diagnosis or operation to recurrence or last follow-up. P values of <0.05 or less were deemed to be statistically significant.

### Ethics approval and consent to participate

All protocols performed in studies involving human participants were reviewed and approved by the Scientific Research and Clinical Laboratory Ethics Committee of the First Affiliated Hospital of Zhengzhou University, China. Informed consent was obtained from all the patients.

## Results

### Bioinformatics analysis of gene expression data and clinical data from TCGA

**Figure 1:**
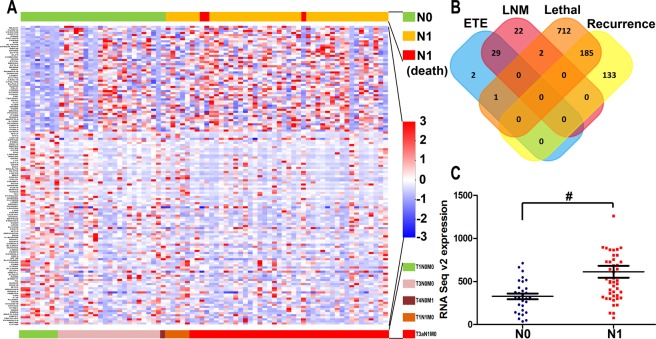
Differentially expressed gene screen. (**A**) Heatmap: A subset of genes whose expression was significantly associated with PTMC lymph node metastasis. (**B**) Venn diagram: Only two genes were overexpressed in both the lymph node metastasis group and the lethal outcome group (*P* < 0.05, fold change >2). MEDAG was one of these genes and exhibited a fold change >1.5 in the recurrence outcome group. The other instances of expression of this gene were so low as to be of no biological significance. (**C**) MEDAG mRNA Seq expression between the LNM-positive and LNM-negative groups (^#^*P* = 0.029).

MEDAG was identified as a differentially expressed gene with biological significance and was significantly high in lethal outcome groups (***P*** < 0.05, fold change >2), the LNM group (***P*** < 0.05, fold change >2) and in the recurrence group(***P*** < 0.05, fold change >1.5). Meanwhile, the differentially expressed genes showed almost no intersection between the poor outcome (recurrence/lethal) group and the aggressive (invasion and metastasis) group (Fig. 1).

### Association between MEDAG expression and clinical pathological characteristics in patients with PTMC

Of the 67 patients, 48 (63%) were female and 19 (37%) were male. Their average age at diagnosis was 45.3 years (range, 26–74 years). ETE of the tumor was found in 6 patients (8%). Initial lymph node metastases were noted in 12 patients (30%). Multicentric tumors were detected in 21 patients (28%). Histologically, 63 (94%) tumors were of the classical type, 3 (4%) were follicular variant of papillary thyroid carcinoma (FVPTC), and 1 (1%) was tall cell variant (TCV). There were 61 patients with stage I/II disease (91%) according to the American Joint Committee on Cancer staging criteria. High MEDAG expression in patients with PTMC was positively correlated with LNM (***P*** = 0.003) and multicenter tumors (***P*** = 0.049). However, no obvious relationship was found with age, gender, non-classical status, extrathyroid invasion, distant metastasis, tumor stage or BRAFv600 mutation (***P*** > 0.05) (Table 1).

**Table 1:** MEDAG expression in PTMC and its relation.

Pathological characteristics	Cases	MEDAG expression		P
		Low (n = 13)	High (n = 54)	
**Age**				
<45	33 (49%)	4	29	0.138
≥45	34 (51%)	9	25	
**Gender**				
Female	48 (72%)	9	39	1
Male	19 (28%)	4	15	
**PTC variants**				
Classcial	62 (93%)	13	49	0.580
Not-classcial	5 (7%)	0	5	
**Extrathyroid invasion**				
No	61 (91%)	13	48	0.609
Yes	6 (9%)	2	4	
**Multicenter tumour**				
No	46 (69%)	12	34	0.049
Yes	21 (31%)	1	20	
**Lymph node metastasis**				
No	44 (66%)	13	31	0.003
Yes	23 (34%)	0	23	
**Distant metastases**				
No	66 (99%)	13	53	1
Yes	1 (1%)	0	1	
**Tumour stage**				
I/II	61 (91%)	11	50	0.329
III/IV	6 (9%)	2	4	
**BRAFv600 mutation**				
No	4 (6%)	0	4	0.579
Yes	63 (94%)	13	50	

### Expression of MEDAG in PTMC

**Figure 2:**
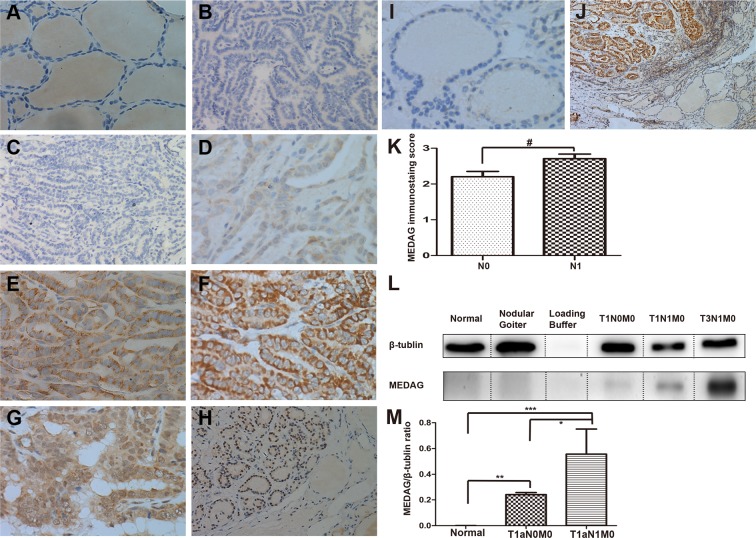
Immunohistochemical and western blot analysis of MEDAG in PTC, para-cancer and nodular goiter tissues: Paraffin-embedded sections were stained using an anti-MEDAG monoclonal antibody as described in the Methods section. Benign tissue sections and negative control samples showing no detectable immunohistochemical staining (**A,B**). Negative(−) or faint(+) cytoplasmic staining was observed in PTC without LNM (**C,D**). Moderate(++) to strong(+++) cytoplasmic staining were visible in LNM cases (**E,F**). Nuclear and/or cytoplasmic staining were observed in FVPTC (**G,H**). Scores for LNM-positive sections are 2.71 (±0.62), compared with LNM-negative 2.20 (±0.98). ^#^P < 0.05, Mann-Whitney Test (**K**). Immunohistochemical staining of MEDAG in nodular goiter (**I**). Immunohistochemical staining of MEDAG PTC and para-cancer tissues (**J**). Ratio of total MEDAG signal to β-tubulin signal in the WB. The data are presented as the mean values ± S.E.M. One-way ANOVA: *P < 0.05; **P, ***P < 0.01 (**M**). Representative images of WB analysis of benign thyroid tissue, nodular goiter and different stages of PTMC (**D**). Original magnification (**A–G**), (**I**): ×400; (**H,J**): ×100. (**L**) The β-Tubulin blots were exposed for 4 s and MEDAG blots exposed for 3 min.

MEDAG staining was absent in the vast majority of para-cancer tissues (64/67, 95.5%) (Fig. 2A). MEDAG expression was detected in 65 out of 67 tumor samples (97%) (Fig. 2B–H). MEDAG staining was either cytoplasmic, nuclear or both. The staining intensity varied from negative to high (Fig. 2C–H). MEDAG expression in nodular goiter and normal tissue was essentially negative as detected by IHC (Fig. 2I, 2J) and WB analysis (Fig. 2L) compared with PTMC at any TNM stage. The expression of MEDAG was greater in LNM samples than in LNM-negative samples (***P*** = 0.042), and there was a distinct difference in expression between PTC and normal tissue (***P*** = 0.001) (Fig. 2L,M).

### Expression of MEDAG in different histological subtypes

**Figure 3:**
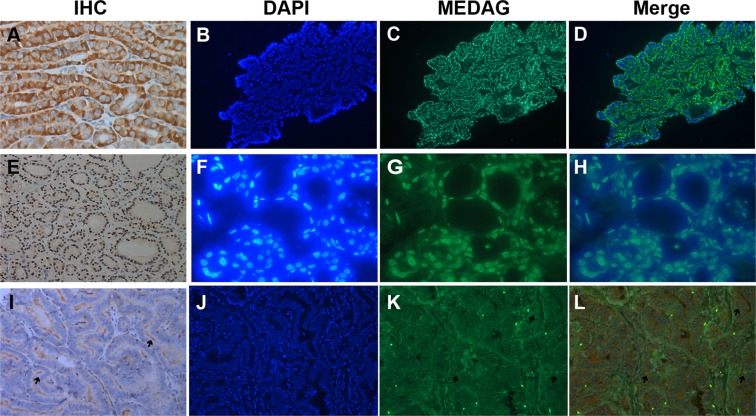
Representative photomicrographs show immunostaining of MEDAG in different subtypes of PTC. Strong cytoplasmic staining of MEDAG in the classical PTMC with a detectable BRAFv600 mutation (**A**), (**C**) and (**D**). Strong nuclear staining of MEDAG in the follicular variant of PTMC without a detectable BRAFv600 mutation (**E**), (**G**) and (**H**). Staining of MEDAG was limited to the margin in tall cell variants PTMC. The black arrow indicates the high expression in the cytoplasm (**I**), (**K**) and (**L**) compared to PPARG (red fluorescent in **L**). Nuclear staining by DAPI (blue fluorescent in **B**, **F** and **J**). MEDAG (green fluorescent in **C**, **D**, **G**, **H**, **K** and **L**). Original magnifications: E × 100; A–D, F–L × 400.

In 62 classical PTC cases, staining was nearly always cytoplasmic (98%) (Figs 2C–F and 3A–D), and only 1 of 62 classical PTCs exhibited both cytoplasmic and nuclear staining (Fig. 2G), which was indicative of the BRAFv600 mutation. MEDAG staining was different in 4 FVPTC cases, in which the BRAFv600 mutation was not detected. Both cytoplasmic and nuclear staining were observed in three cases, and one case exhibited only nuclear staining (Figs 2H and 3E–H). Nuclear or both cytoplasmic and nuclear staining were associated with the FVPTC histopathological subtype (***P*** = 0.025). In tall cell variant PTC, MEDAG staining was observed in the cytoplasm but was limited to the margin (Fig. 3I–L).

**Figure 4:**
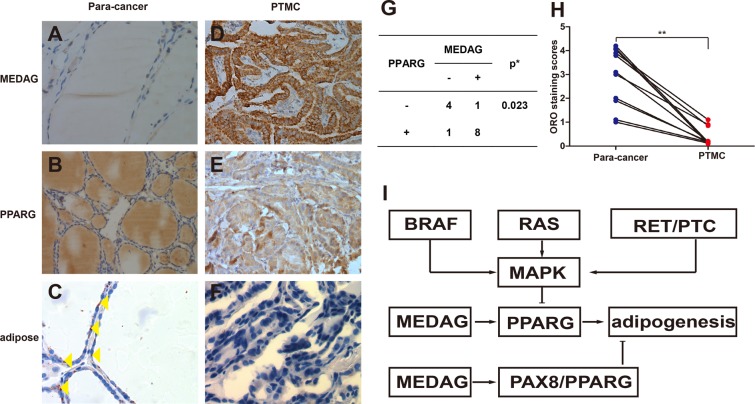
The representative photomicrographs show immunostaining of MEDAG, PPARG and ORO staining of normal thyroid and thyroid carcinomas. In normal thyroid samples, MEDAG staining was negative (**A**), and in some cases, PPARG immunostaining was negative (**B**), but ORO staining was positive (**C**). Lipid droplets were rarely found in PTMC (**F**), even though MEDAG and PPARG were highly expressed (**D**,**E**). There was a statistically significant association between the MEDAG over expression and PPARG expression (**G**). The ORO staining scores were different between paracancer and PTMC samples (*P* = 0.00009) (**H**). The potential for metabolic reprogramming was observed in PTMC (**I**). Yellow triangles show the lipid droplets in the cytoplasm. *Fisher’s Exact Test, **Wilcoxon test. Original magnifications: B × 100; A,C–F × 400.

### Association between MEDAG expression and adipogenesis in PTMC

Previous *in vivo* and *in vitro* studies have confirmed the function of MEDAG in adipogenesis. We detected lipid droplets by Oil Red O (ORO) staining in PTMC compared with mesentery samples. MEDAG expression was also detected at the same time. However, few lipid droplets were detected in PTMC, even when MEDAG was highly expressed (Fig. 4D,F), compared with mesentery and normal thyroid tissue (***P*** = 0.00009) (Fig. 4A,C,H). Additionally, there was a statistically significant association between the MEDAG over expression and PPARG expression in PTMC (***P*** = 0.023) (Fig. 4D,E,G). The high expression of MEDAG in PTMC with adipogenesis may indicate that the downstream process of adipogenesis was disrupted or hindered.

### Pathway and differential expression analysis of PTMC metabolism in adipogenesis

**Figure 5:**
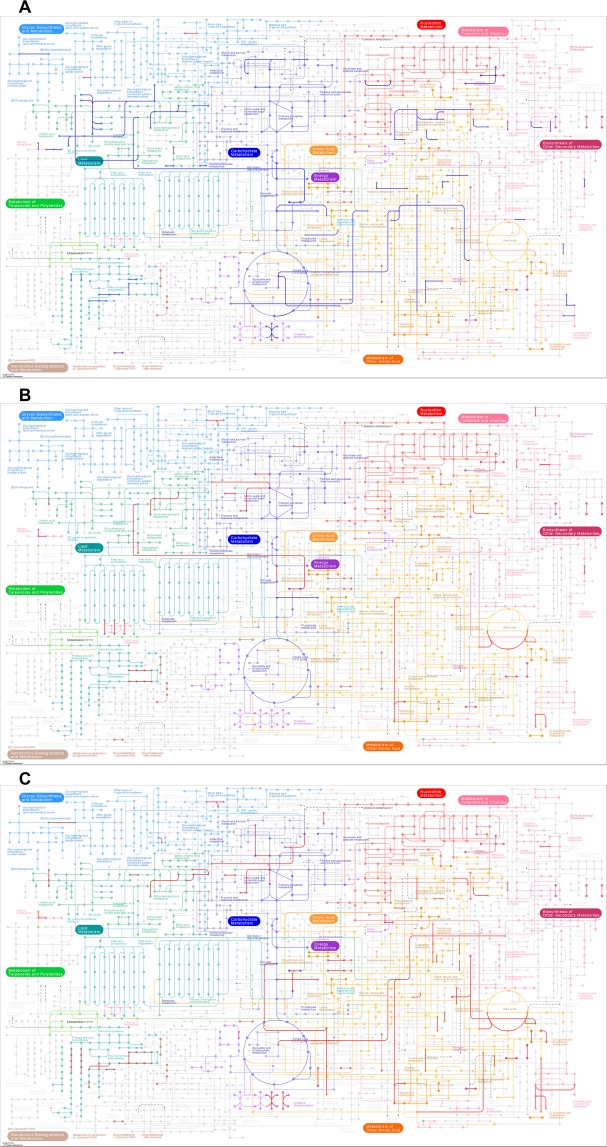
The metabolic pathways of differentially expressed genes (P < 0.05, fold change >2). Pathways enriched in up-regulated genes are indicated by red lines, and pathways enriched for down-regulated genes are indicated by blue lines. (**A**) Differential expression states for genes enriched in the LNM group. (**B**) Differential expression states for genes enriched in the poor outcome group. (**C**) Differential expression states for genes enriched in the lethal outcome group^21^.

To explore metabolic reprogramming and its role in PTMC, we performed the the categorization of annotations of the differentially expressed genes (***P*** < 0.05, fold change >2) in KEGG metabolic pathways, and included fatty acid metabolism, lipid metabolism, sterol biosynthesis, glycolysis and the citrate cycle (TCA cycle). The main metabolic pathways in which differentially expressed genes are enriched are highlighted by colored lines on the KEGG metabolism pathway diagram (Fig. 5). Genes with downregulated expression were enriched in fatty acid biosynthesis, triacylglycerol biosynthesis, acylglycerol degradation, and ceramide biosynthesis pathways, etc. in the LNM group(Fig. 5A). In the poor outcome group, the genes with upregulated differential expression were involved in fatty acid biosynthesis, ketone body biosynthesis, triacylglycerol biosynthesis, acylglycerol degradation, ceramide biosynthesis, sphingosine biosynthesis, and sterol biosynthesis pathways, etc. (Fig. 5B). In the lethal outcome group, the genes with upregulated expression were enriched in triacylglycerol biosynthesis, acylglycerol degradation, phosphatidylcholine biosynthesis, glycosphingolipid biosynthesis, bile acid biosynthesis, and steroid hormone biosynthesis pathways. Genes with downregulated expression were enriched in carbon fixation, glycolysis, and citrate cycle pathways (TCA cycle) (Fig. 5C).

### Survival analysis

**Figure 6:**
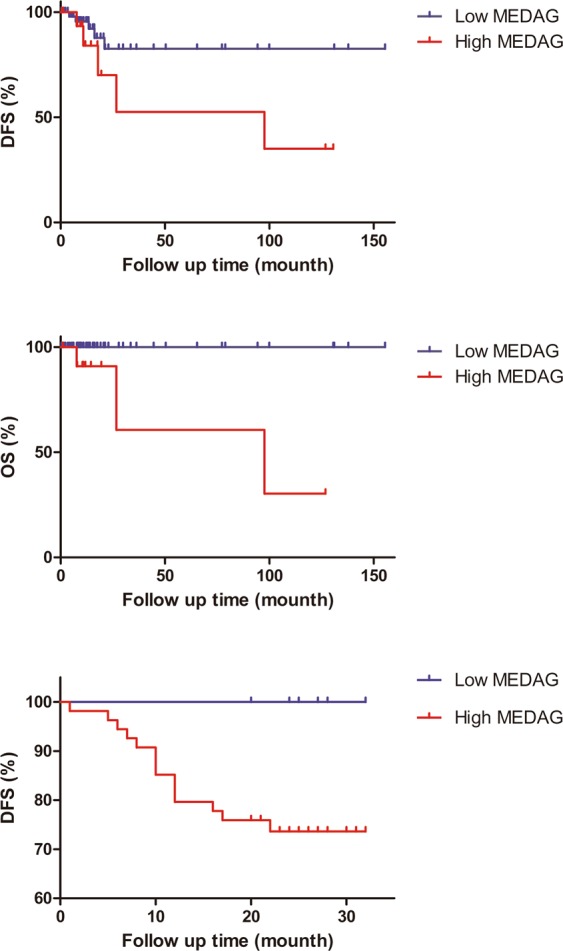
High expression of MEDAG was associated with poor overall survival and poor disease-free survival. Kaplan-Meier curves for overall survival (**A**) and disease-free survival (**B**) in PTMC patients from the TCGA database. (**C**) In our cohort, disease-free status is different, as estimated by the Kaplan–Meier method and compared by the log-rank test (P < 0.05).

To investigate the prognostic value of MEDAG for PTMC, we evaluated the association between MEDAG expression and survival duration using a Kaplan–Meier analysis based on data from both the TCGA database (Fig. 6A,B) and on our cohort (Fig. 6C). In our study, the median duration of follow-up was 9.6 months (range, 1–32 months). Locoregional recurrence was noted in patients (20.9%), while 1 (1.5%) patient developed metastases; no patients deaths were reported. The log-rank test showed that the DFS was different between the group of PTMC patients with high MEDAG expression and the group with low MEDAG expression (*P* = 0.0495). In short, the MEDAG over-expression group had a shorter DFS duration. The result in our cohort was similar to that from the TCGA database. In our cohort, MEDAG over-expression was associated with poorer DFS (OR = 1.350, 95% CI 1.153–1.581, *P* = 0.039), and TNM stage was associated with poorer DFS (OR = 4.352, 95% CI 1.090–17.378, *P* = 0.028) as well. Other variables, including age ≥45 (OR = 0.670, 95% CI 0.204–2.197, *P* = 0.507), male sex (OR = 2.308, 95% CI 0.675–7.892, *P* = 0.176), ETE (OR = 2.042, 95% CI 0.334–12.488, *P* = 0.432), LNM (OR = 2.308, 95% CI 0.675–7.892, *P* = 0.176), multifocality (OR = 1.900, 95% CI 0.563–6.407, *P* = 0.296), and BRAF mutation (OR = 0.780, 95% CI 0.075–8.130, *P* = 0.835), were not associated with poorer DFS.

## Discussion

In this study, we initially investigated the potential prognostic biomarkers of PTMC. To identify the molecular aberrations associated with progression and clinical characteristics in PTMCs, we downloaded gene expression data and clinical data from TCGA, and examined the differentially expressed genes. We then used IHC to verify the results in our cohort and confirm that overexpression of  MEDAG was related to LNM and poor prognosis in PTMC. Finally, the effects on Oil Red O staining and pathway analysis of differential expression genes indicated that the biological process of adipose tissue synthesis mediated by MEDAG involves metabolic reprogramming in PTMC. In summary, our results imply that MEDAG could be a potential biomarker of PTMC. Our findings may be useful in directing PTMC treatment to affect clinical outcomes, especially in screening low risk PTMC patients with active surveillance to avoid immediate surgery.

This study showed for the first time that MEDAG expression plays a role in cancer. Our result show that MEDAG localized to the cytoplasm in most classical PTMCs, but few lipid droplets were found in PTMC compared with para-cancer or adipose tissue, even when MEDAG was highly expressed in PTMC. However, previous investigations indicate that MEDAG plays an important role in carbohydrate and lipid metabolism in the cytoplasm and acts as a regulator of the transcription factor PPARG. To explain this phenomenon, we hypothesize that PPARG could be depressed and down-regulated by the MAPK signaling pathway as previously reported^22–24^. This signaling pathway is usually activated by RAS mutation, BRAF mutation, or RET/PTC rearrangement in PTC^25,26^. This indicated that the biological process of adipose tissue synthesis mediated by MEDAG involves metabolic reprogramming^27–29^ (Fig. 4K). Furthermore, since MEDAG is also significantly differentially expressed in healing gingiva^20^, we believe the overexpression in PTMC could be in line with the hypothesis–“Tumors: wounds that do not heal”^30^.

In practice, PTMC is more likely to be considered as a clinical subset of PTC with an indolent course, rather than a histological subtype. No animal model or cell lines  have been reported as suitable for PTMC research. For example, TERT promoter mutation, a prognostic genetic marker for thyroid cancer^31–33^, is universal in almost all types of thyroid cancer cell lines^34^, but is a rare genetic event in PTMC^35,36^. To accurately reflect the lipid metabolism characteristics and mechanisms underlying the distinct prognoses of invasive PTMC, in which MEDAG may be involved, differentially expressed gene analysis was carried out by KEGG Mapper. As shown in Fig. 5, the metabolic features in the LNM group may lead to cytomembrane flexibility to overcome hurdles for cell motility and initiation of ER stress. The former is thought to be necessary for metastasis^37,38^, and the latter may inhibit PTMC cells growth^39–41^. However, the lethal prognosis group seemed to easily tolerate ER stress, and the poor prognosis group exhibited characteristics of lipid accumulation, which was also reported in the cribriform variant of PTC^42^. Additionally, we were surprised by the results of the Venn diagram, which shows that the greatest difference overexpressed and underexpressed genes (data not shown) was between the poor outcome group and aggressive clinical characteristics group. This suggests that the poor prognosis group may be a special subset in PTMC in which tumor progression and metastasis may be distinct processes^38^.

The localization of MEDAG to the nucleus was also observed in FVPTC, especially in BRAFv600 mutant-negative samples in our study. FVPTC is considered to have fewer BRAFv600 mutations but more PAX/PPARG rearrangements, which can also be combined with other genetic mutations. In PTC with PAX/PPARG rearrangement, abnormal hyperactivation of the PAX8 promoter leads to increased expression of the fusion protein^43^. Therefore, we hypothesized that MEDAG, which acts as a positive transcription factor, may be restricted within PTC’s nuclei with PAX/PPARG rearrangement.

In our study, we observed a trend towards decreased expression and localization of MEDAG in TCV. Although it is unknown whether the cause of this tendency is protein or mRNA degradation, it may be beneficial to TCV cancer cells during cellar stress. Similar to anaplastic thyroid carcinoma (ATC), TCV exhibits a higher proportion of TP53 mutation than does classical PTC, and both of them have poorer prognosis^44,45^. This phenomenon may be in line with the metabolic feature of the poor prognosis group and the “second hit” hypothesis^46^. Additionally, we suspect that there is a potential mechanism by which the reducing in MEDAG would free the tumor from inertia in non-PTMC.

In our resaerch, we investigated the potential prognostic biomarkers of PTMC and their underlying mechanisms using clinical samples and by bioinformatics methods, as no animal model or cell lines have been reported suitable for PTMC research. Thus, additional rationally designed experiments need to be carried out *in vivo* and *in vitro*. And we also observed that the K-M curve is not sufficient with regards to DFS (Fig. 6A,6C). This partly rely on differences in the definition of DFS, expression platform or that follow-up time is not long enough for this clinically indolent tumor. As classical PTC accounts for the majority of PTC in both the TCGA database and our cohort, the biomarker analysis was almost entirely based on classical PTMC. So, we speculate that various histological subtypes of PTC should be taken into account, when the MEDAG expression will be evaluated in PTMC FNA samples.

## Conclusion

In summary, our results are useful for diagnosing PTMC and providing information about prognosis and the likelihood of recurrence after surgery. MEDAG expression could be a novel prognostic marker for PTMC patients. Additional studies are needed in non-PTMC cases as well as *in vivo* and *in vitro* studies.

## Supplementary information

supplementary information

## Data Availability

Microarray data and clinical data referred to in this article can be found in TCGA. Please contact author for all other data requests.
