# Supplementary material for: Evaluation of MEDAG gene expression in papillary thyroid microcarcinoma: associations with histological features, regional lymph node metastasis and prognosis
Source: Sci Rep. 2019 Apr 9;9:5800. doi: 10.1038/s41598-019-41701-4 (PMC6456583; doi:10.1038/s41598-019-41701-4)
Supplement: Supplementary file 1 — supplementary information [file 41598_2019_41701_MOESM1_ESM.pdf]

Evaluation of MEDAG gene expression in papillary thyroid microcarcinoma: associations with histological features, regional lymph node metastasis and prognosis

Yang Song[1,2#], Li-jun Fu[1#], Hong-ting Li[1], Xin-guang Qiu[1*].

Yang Song, Department of thyroid surgery, The First Affiliated Hospital of Zhengzhou University, Construction of Road No.1, Zhengzhou, P.R.China. And Department of Oncological Surgery, Xinyang Cental Hospital, Siyi of Road No.1, Xinyang, P.R.China

Li-jun Fu, Department of thyroid surgery, The First Affiliated Hospital of Zhengzhou University, Construction of Road No.1, Zhengzhou, P.R.China

Hong-ting Li, Department of thyroid surgery, The First Affiliated Hospital of Zhengzhou University, Construction of Road No.1, Zhengzhou, P.R.China

Xin-guang Qiu, Department of thyroid surgery, The First Affiliated Hospital of Zhengzhou University, Construction of Road No.1, Zhengzhou, P.R.China

[#]Contributed equally.

Correspondence to: Xinguang Qiu. M.D., Ph.D., Department of thyroid surgery, The First Affiliated Hospital of Zhengzhou University, Construction of Road No.1, Zhengzhou, P.R.China. Tel: +86 13803710710. E-mail: tjky2012@126.com.

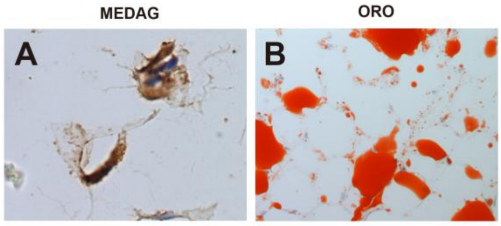

Supplemental Figures: Figure S1: Mesentery samples, as positive controls, were positive for both IHC and ORO staining (A), (B).A×400 and magnified, B×400.
